# Supplementary material for: The genome sequence of the Scarce Copper, Lycaena virgaureae (Linnaeus, 1758) (Lepidoptera: Lycaenidae)
Source: Wellcome Open Res. 2025 Aug 11;10:434. [Version 1] doi: 10.12688/wellcomeopenres.24748.1 (PMC12432500; doi:10.12688/wellcomeopenres.24748.1)
Supplement: Peer review history

# Reviewer report: Reviewer response for version 1

**DOI:** 10.21956/wellcomeopenres.27265.r129693

**Published:** 2025-09-12

**Authors:** Melissa Toups, Zhaobo Hu

**Affiliations:** 1 University of Louisiana at Lafayette, Lafayette, Louisiana, USA; 2 Biology, University of Louisiana at Lafayette Department of Biology, Lafayette, Louisiana, USA

The authors present a chromosome-level assembly of the Scarce Copper, *Lyceana virgaureae*. The methods are well-described, state-of-the-art, and replicable. I have two comments.

 First, the authors identify the Z chromosome simply using homology with a closely related species instead of independent verification using coverage and/or sex-linked polymorphism. As the Z is well-conserved in Lepidoptera, I expect they are likely correct, but technically this should probably be referred to as the "putative Z".  

 Second, an annotated gene set would significantly improve the utility of this assembly for other researchers.

Are sufficient details of methods and materials provided to allow replication by others?

Yes

Is the rationale for creating the dataset(s) clearly described?

Yes

Are the datasets clearly presented in a useable and accessible format?

Yes

Are the protocols appropriate and is the work technically sound?

Yes

Reviewer Expertise:

sex-chromosome evolution, evolutionary genetics, genomics

We confirm that we have read this submission and believe that we have an appropriate level of expertise to confirm that it is of an acceptable scientific standard.

# Reviewer report: Reviewer response for version 1

**DOI:** 10.21956/wellcomeopenres.27265.r129699

**Published:** 2025-09-08

**Authors:** Nitin Ravikanthachari

**Affiliations:** 1 University of Montana, Missoula, Montana, USA

The manuscript provides a new genome resource for the butterfly- Lycaena virgureae. The authors have done a thorough job to sequence the genome and the metrics reported reflect that the genome is of high quality. This will be a valuable resource in the coming years for evolutionary biologists and in the applied field of conservation biology.

 Overall, the manuscript is of great quality. However, I would like the authors to conduct genome and functional annotation to improve the manuscript and to set the standard for future genome reports. The authors could use an automated pipeline such as BRAKER2 and EggNOG for genome and functional annotation or use another manual/automated pipeline of their own liking. 

 I believe that adding this information would improve the manuscript and would benefit a broader range of scientists.

Are sufficient details of methods and materials provided to allow replication by others?

Yes

Is the rationale for creating the dataset(s) clearly described?

Yes

Are the datasets clearly presented in a useable and accessible format?

Yes

Are the protocols appropriate and is the work technically sound?

Yes

Reviewer Expertise:

Genomics, evolutionary genetics, host-microbe interactions, plant-animal interactions, population genomics.

I confirm that I have read this submission and believe that I have an appropriate level of expertise to confirm that it is of an acceptable scientific standard, however I have significant reservations, as outlined above.

# Reviewer report: Reviewer response for version 1

**DOI:** 10.21956/wellcomeopenres.27265.r129698

**Published:** 2025-08-21

**Authors:** Yang Mei

**Affiliations:** 1 Jilin Agricultural University, Changchun, Jilin, China

This manuscript reports the high-quality assembly of a butterfly, the  Scarce Copper, *Lycaena virgaureae*. And the protocols and methods were described well and clearly. So, I agree to index this paper.

 But I think the genome annotation information is necessary for related researchers, so maybe the OGS should be made available.

Are sufficient details of methods and materials provided to allow replication by others?

Yes

Is the rationale for creating the dataset(s) clearly described?

Yes

Are the datasets clearly presented in a useable and accessible format?

Yes

Are the protocols appropriate and is the work technically sound?

Yes

Reviewer Expertise:

insect genomics

I confirm that I have read this submission and believe that I have an appropriate level of expertise to confirm that it is of an acceptable scientific standard.
